# Laparoscopic Laser Speckle Contrast Imaging Can Visualize Anastomotic Perfusion: A Demonstration in a Porcine Model

**DOI:** 10.3390/life12081251

**Published:** 2022-08-16

**Authors:** Aurelia Wildeboer, Wido Heeman, Arne van der Bilt, Christiaan Hoff, Joost Calon, E. Christiaan Boerma, Mahdi Al-Taher, Nicole Bouvy

**Affiliations:** 1 Department of Surgery, Maastricht University Medical Center, 6229 HX Maastricht, The Netherlands; 2 University Campus Fryslân, University of Groningen, 8911 CE Leeuwarden, The Netherlands; 3 Department of Surgery, University Medical Centre Groningen, 9713 GZ Groningen, The Netherlands; 4 LIMIS Development BV, 8934 AD Leeuwarden, The Netherlands; 5 Department of Surgery, Medical Centre Leeuwarden, 8934 AD Leeuwarden, The Netherlands; 6 ZiuZ Visual Intelligence BV, 8401 DK Gorredijk, The Netherlands; 7 Department of Intensive Care, Medical Centre Leeuwarden, 8934 AD Leeuwarden, The Netherlands

**Keywords:** laser speckle contrast imaging, anastomotic leakage, perfusion assessment, laparoscopic surgery, real-time imaging, image-guided surgery

## Abstract

Background: Intestinal resection causes inevitable vascular damage, which cannot always be seen during an intraoperative clinical assessment of local intestinal perfusion. If left unaltered, impaired perfusion can lead to complications, such as anastomotic leakage (AL). Therefore, we demonstrate the use of a novel laparoscopic laser speckle contrast imaging (LSCI)-based approach in order to assess local intestinal perfusion during the construction of intestinal anastomoses. Methods: Three segments were isolated from the small intestine of a pig, while the perfusion of each was compromised by coagulating 7–8 mesenteric arteries. Both clinical assessments and LSCI were used to detect the induced perfusion deficits and to subsequently guide a transection in either a well perfused, marginally perfused, or poorly perfused tissue area within the segment. Bowel ends were then utilized for the creation of three differently perfused anastomoses: well perfused/well perfused (anastomosis segment 1), well perfused/poorly perfused (anastomosis segment 2), and poorly perfused/poorly perfused (anastomosis segment 3). After construction of the anastomoses, a final perfusion assessment using both clinical assessment and LSCI was executed in order to evaluate the vascular viability of the anastomosis. Results: Laparoscopic LSCI enabled continuous assessment of local intestinal perfusion and allowed for detection of perfusion deficits in real time. The imaging feedback precisely guided the surgical procedure, and, when evaluating the final anastomotic perfusion, LSCI was able to visualize the varying degrees of perfusion, whereas standard clinical assessment yielded only minor differences in visual appearance of the tissue. Conclusions: In this technical note, we demonstrate a novel LSCI-based approach for intraoperative perfusion assessment. With its ability to continuously visualize perfusion in real time, laparoscopic LSCI has significant potential for the optimization of anastomotic surgery in the near future.

## 1. Introduction

Intestinal resection causes inevitable vascular damage, which is not always evident during an intraoperative clinical assessment of local intestinal perfusion. If left unaltered, impaired perfusion can lead to anastomotic healing complications such as anastomotic leakage (AL) [1]. Owing to its detrimental effect on both short and long-term outcomes (e.g., increased 30-day mortality risk, worsened oncological prognosis), AL is also one of the most severe possible complications of gastrointestinal tract surgery. A significant percentage of patients undergoing restorative intestinal surgery develop AL, leading to both considerable morbidity and mortality [2,3,4]. Impaired perfusion is one of the key factors implicated in anastomotic failure. Ensuring adequate perfusion is an important part of a multidimensional approach to gradually improving the overall outcome in restorative intestinal surgery.

In recent years, the use of intraoperative image-guided surgery techniques has become increasingly popular as a tool used to avoid excessive vascular damage. Several techniques, such as indocyanine green near-infrared fluorescence imaging (ICG-NIRF), have been developed to improve intraoperative perfusion assessment, as well as to navigate intestinal resections. Compared with the traditional visual inspection for subjective signs of bowel viability (e.g., mucosal and serosal color, pulsatile bleeding from marginal arteries, bowel peristalsis), these techniques have the potential to be a transformative tool in the surgical field [5,6,7]. Despite much research and development, fluorescence imaging is still facing fundamental challenges that diminish its potential to become the ideal bowel viability testing technique. Albeit effective, this imaging method poses several inconveniences linked to the needed dye. The use of dyes limits both the length of real-time imaging and the repeatability of the assessment [8,9]. PerfusiX-Imaging (LIMIS Development BV, Leeuwarden, The Netherlands) is a dye-free method that could simplify, and thus speed up, intraoperative perfusion assessment. Dye-free imaging means there is no interruption of the surgical workflow due to both dye injection and to instantaneous imaging when deemed necessary. There is no wash-out effect, resulting in continuous and repeated perfusion measurements. Furthermore, no change is required regarding the surgeons’ tools, as the system is designed to be compatible as an add-on to laparoscopic imaging systems.

We hypothesize that the use of laparoscopic laser speckle contrast imaging (LSCI) PerfusiX-Imaging has the potential to detect perfusion differences in intestinal anastomoses, as we have already demonstrated that LSCI is capable of identifying ischemic areas on the large intestine in a standard laparoscopic setup without constructing anastomoses [10]. In this technical note, we demonstrate the use of a novel laparoscopic LSCI device by purposely creating perfusion-deficient anastomoses.

## 2. Materials and Methods

This technical demonstration was performed at the central animal facility of Maastricht University (Maastricht, The Netherlands). The animal was treated in compliance with the regulations of the Dutch legislation for animal research, and a protocol approved by the local animal ethics committee was followed.

### 2.1. Animal

One female Dutch landrace pig, weighing 35.0 kg, was used. After an acclimatization period in the animal keeping facility and a 24-h fast with free access to water, premedication was given, which consisted of an intramuscular injection of zolazepam/tiletamine 6 mg/kg (Virbac, Barneveld, The Netherlands) and thiopental 10 mg/kg (Panpharma SA, Trittau, Germany). Anesthesia consisting of sufentanyl 0.01 mg/kg/h (Hameln Pharma GmbH, Hameln, Germany), propofol 9 mg/kg/h (B. Braun Melsungen AG, Melsungen, Germany), and midazolam 1 mg/kg/h (Aurobindo, Baarn, The Netherlands) was induced via an intravenous injection. After intubation, the pig was mechanically ventilated, and, whenever necessary, anesthesia was intensified using an additional administration of sufentanyl and propofol. At the end of the procedure, the animal was euthanized with a lethal dose of pentobarbital 200 mg/kg (AST Farma, Oudewater, The Netherlands).

### 2.2. Surgical Procedure

In order to create three differently perfused anastomoses, a pneumoperitoneum was established through supraumbilical placement of a 10-mm port, for a laparoscope (OTV-S200, Olympus, Hamburg, Germany) with carbon dioxide gas at a pressure of 8 mmHg. Additionally, three working ports (5-mm) were inserted. Once sufficient access to the peritoneum and small bowel was achieved, three intestinal segments were identified and isolated. Subsequently, windows were created in the mesentery of each segment, after which 7 to 8 peripheral vessels were dissected using an energy device (Thunderbeat, Olympus, Hamburg, Germany). After perfusion limitation, LSCI was used to guide the surgeon within the sections of well and poorly perfused tissue. A side-to-side stapled anastomosis was created using a laparoscopic stapling device (Endo GIA™ with 60 mm reload, Medtronic, Minneapolis, MN, USA). The stapler defects were then closed with a continuous suture (Prolene 3/0, Ethicon™, Johnson & Johnson Health Care Systems Inc., Piscataway, NJ, USA). Both a clinical and an LSCI assessment of local intestinal perfusion were performed before, during, and after perfusion limitation, intestinal transection, and creation of the anastomoses, respectively.

### 2.3. Laparoscopic LSCI Setup and Data Acquisition

**Figure 1:**
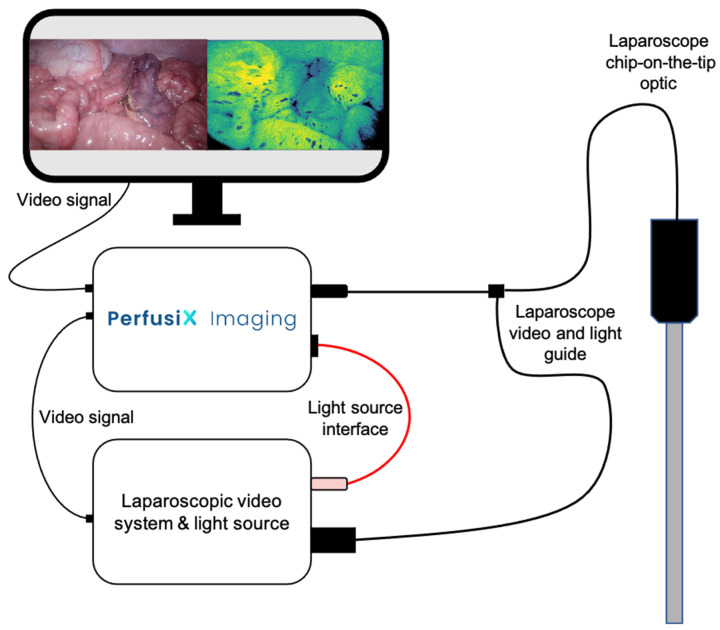
A schematic figure of PerfusiX-Imaging in conjunction with a laparoscopic video system and light source. The light guide of the chip-on-the-tip laparoscope is plugged into the PerfusiX-Imaging. The PerfusiX-Imaging light source interface is connected to the laparoscopic light source. The video signal is received using an SDI output. The PerfusiX-Imaging perfusion images are displayed on the available monitors in the operating room in real time.

The PerfusiX-Imaging (LIMIS Development BV, Leeuwarden, The Netherlands) device was used to acquire laparoscopic LSCI images [10]. PerfusiX-Imaging is a laparoscopic perfusion imager that is designed as an add-on for a laparoscopic system (Figure 1). In this case, an Olympus laparoscopic video system (OTV-S200, Olympus, Hamburg, Germany) and a 30-degree laparoscope (EndoEye, Olympus, Hamburg, Germany) were used. LSCI is a dye-free, non-contact, and non-invasive technique that uses laser light to detect the motion of red blood cells in real time [11]. It instantaneously and continuously provides real-time 2D perfusion maps. LSCI utilizes a low-powered laser light source to illuminate the tissue of interest. The interference then forms a pattern on the camera sensor, which is referred to as a speckle pattern. This pattern changes in accordance with the movement of underlying red blood cells at a rate that corresponds to the amount of blood flow. Hence, it is this blurring, or loss in contrast, which is quantified as laser speckle perfusion units (LSPU) [11]. The LSPU is inversely correlated to speckle contrast K. Higher LSPU values correspond to better tissue perfusion as compared to lower LSPU values. The recorded images are converted into grayscale images, which are used to determine the LSPU by calculating the ratio of the standard deviation divided by the mean intensity of the pixels within the sliding window (Equation (1)). Images were acquired at 20 frames per second and analyzed using spatial LSCI with a window size of 7 × 7 pixels (PerfusiX-Imaging software suite, LIMIS Development BV, Leeuwarden, The Netherlands).

(1) $$K=\frac{\sigma }{\langle I\rangle }$$

LSCI is a fast and full-field imaging technique that can image large surfaces without the need for a contrast agent. Non-invasive subsurface perfusion measurements are characterized by a high spatial and temporal resolution [11]. This device houses a red laser and allows for instant switching between conventional white light and laser light for the duration of the perfusion measurement. The laser was connected to the laparoscope using the optical fiber attached to the EndoEye. The 2D perfusion maps were directly available in the operating room and were displayed for the surgeons to see in a side-by-side mode. Camera exposure time was 20 milliseconds for all measurements. The aperture could not be determined; however, a constant speckle/pixel ratio higher than one was maintained, satisfying the Nyquist criterion [12].

## 3. Results

### 3.1. Animal and Surgical Procedure

The surgical procedure was performed without any complications or adverse events.

### 3.2. LSCI Perfusion Assessment during Anastomotic Construction

**Figure 2:**
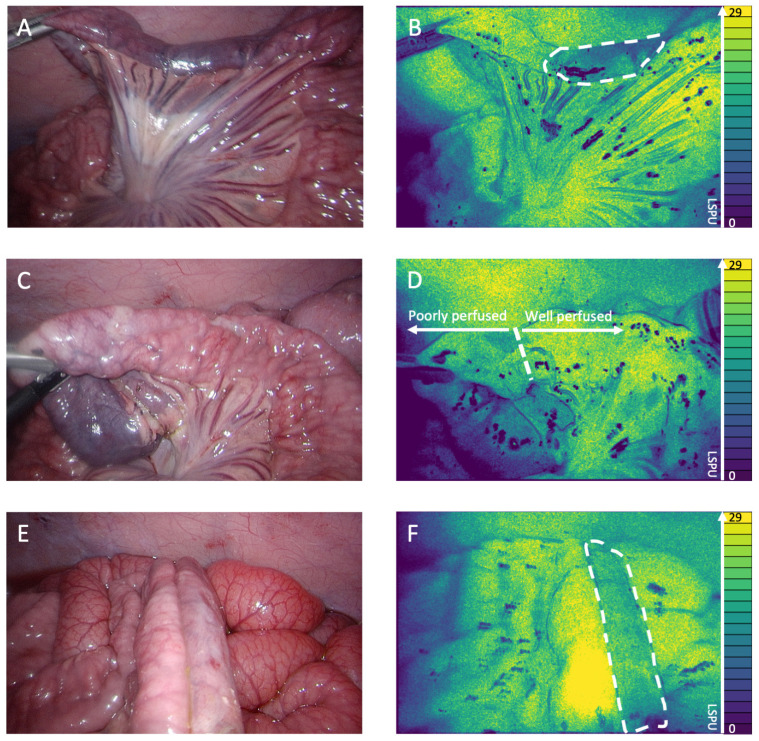
(**A**) White light image of the perfusion deficit. (**B**) 2D perfusion map generated by the PerfusiX-Imaging system (LIMIS Development BV, Leeuwarden, The Netherlands) of the perfusion deficit. The ischemic tissue is highlighted by white dotted lines. The perfusion is displayed in laser speckle perfusion units (LSPU) (A.U.). (**C**) White light image of the transection location. (**D**) 2D perfusion map generated by the PerfusiX-Imaging system of the transection location. The watershed area is highlighted. (**E**) White light image of the well/poorly perfused anastomosis. (**F**) 2D perfusion map generated by the PerfusiX-Imaging system of the well/poorly perfused anastomosis. The poorly perfused tissue is highlighted by white dotted lines.

Real-time visualization of local intestinal microperfusion was achieved in all three stages of anastomotic creation (perfusion limitation, intestinal transection, and anastomotic construction). The 2D perfusion maps allowed us to instantaneously detect the induced perfusion deficits (Figure 2A,B) and make a clear distinction between adequately and poorly perfused tissue regions, which precisely guided intestinal transection (Figure 2C,D) and anastomotic construction (Figure 2E,F), as can be seen in Supplementary Video S1.

### 3.3. LSCI Perfusion Assessment of Anastomoses

**Figure 3:**
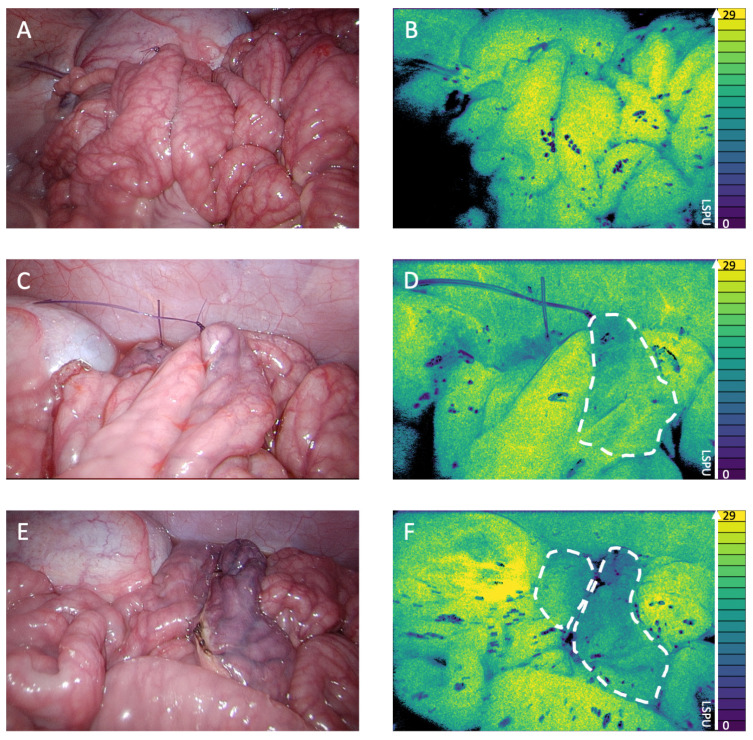
(**A**) White light image of the well-well perfused anastomosis. (**B**) 2D perfusion map generated by the PerfusiX-Imaging system (LIMIS Development BV, Leeuwarden, The Netherlands) of the well-well perfused anastomosis. Black areas are underexposed, and the ischemic tissue is highlighted by white dotted lines. The perfusion is displayed in laser speckle perfusion units (LSPU) (A.U.). (**C**) White light image of the well-poor perfused anastomosis. (**D**) 2D perfusion map generated by the PerfusiX-Imaging system of the well-poor perfused anastomosis. (**E**) White light image of the poor-poor perfused anastomosis. (**F**) 2D perfusion map generated by the PerfusiX-Imaging system of the poor-poor perfused anastomosis.

Figure 3 shows the 2D perfusion images displayed in Viridis false colors [13]. The LSCI perfusion images displayed clear demarcation lines between well perfused and poorly perfused tissue in real time, which correspond to the visual tissue discoloration. Figure 3A (conventional white light) and Figure 3B (LSCI perfusion image) display the well-well perfused anastomotic segment. The well-poor perfused anastomotic segment can be seen in Figure 3C (white light) and Figure 3D (perfusion image), with the ischemic area, as indicated by the 2D perfusion map, highlighted in Figure 3D. The right side of the anastomosis is clearly less perfused than the left side. The poor-poor perfused anastomosis segment is found in Figure 3E (white light) and Figure 3F (perfusion image); both segments showed ischemia, which is more evident on the right part of the anastomosis.

## 4. Discussion

In this technical note, we demonstrate the use of a novel laparoscopic LSCI-based approach for intraoperative perfusion assessment of the anastomosis during intestinal surgery. By creating three variously perfused anastomoses, we were able to demonstrate different perfusion states of an intestinal anastomosis. The detection of impaired perfusion around the anastomosis, as demonstrated in this manuscript, could aid surgeons in relocating to an intestinal segment with more optimal perfusion. This technology allows surgeons to perform intraoperative perfusion imaging prior to, during, and after the construction of anastomoses, with the aim of ensuring the most favorable perfusion state. The perfusion measurements were captured in real time and were established without the use of fluorescent dyes. The imaging feedback precisely guided transections through well perfused, marginally perfused, and poorly perfused tissue, which was confirmed once again by the 2D perfusion images after construction of the anastomoses. This additional PerfusiX-Imaging-derived visual feedback could further the improvement of substantiated clinical decision making during intestinal surgery. However, since this technical note is solely focused on the feasibility of perfusion measurements and the detection of perfusion differences at the anastomosis, further studies with respect to relevant clinical outcomes (e.g., anastomotic leakage, postoperative complication rates) and validation studies (e.g., comparison with indocyanine green, comparison with local lactate levels and pathology) should be performed in order to determine the added clinical value. These larger studies could additionally help determine LSPU ischemia threshold values based on pathology. This value could be envisioned as a percentage of the maximum perfusion in healthy tissue as compared to the perfusion surrounding the anastomosis.

Although this technical note highlights the potential of laparoscopic LSCI, some limitations regarding this demonstration need to be addressed. Despite the performance of multiple demonstrations using three different intestinal segments, only one animal was used. The results of this study should therefore be treated with prudence, as they are limited and do not allow for generalized conclusions. Nevertheless, the aim of this technical note is to demonstrate the potential of this technique when it comes to imaging various anastomotic perfusion states, which justifies the current setup and which respects the directives on animal experimentation (Replacement, Reduction, Refinement) [14].

Altogether, this technical demonstration shows the potential of LSCI-based perfusion imaging, both in order to optimize restorative intestinal surgery and to improve clinical outcomes in the future.

## Data Availability

Not applicable.

## Supplementary Materials

The following supporting information can be downloaded at: https://www.mdpi.com/article/10.3390/life12081251/s1, Video S1: Laparoscopic LSCI demonstration.
